# Supplementary material for: MetaRibo-Seq measures translation in microbiomes
Source: Nat Commun. 2020 Jun 29;11:3268. doi: 10.1038/s41467-020-17081-z (PMC7324362; doi:10.1038/s41467-020-17081-z)
Supplement: Supplementary file 10 — Supplementary Data 7 [file 41467_2020_17081_MOESM10_ESM.zip › File2/Confidence_VeryHigh_Taxonomy/32984_out.krona.html]

Javascript must be enabled to view this page.

members
magnitude
magnitudeUnassigned
count
unassigned
taxon
rank

32984\_out

23

23
2
superkingdom

1239
phylum
23

23
class
186801

23
order
186802

22
family

SRS144135\_contig\_number\_15228
1
186803

1
genus
841


SRS048164\_contig\_number\_33359
species
360807
1

1
genus
1506553


SRS024075\_contig\_number\_12618
species
1531
1

1
genus
28050


SRS077502\_contig\_number\_13781
species
28052
1

18
39491
species

SRS014235\_contig\_number\_49825SRS015578\_contig\_number\_27287SRS015794\_contig\_number\_contig-100\_197.90031SRS016203\_contig\_number\_contig-100\_363.69641SRS019286\_contig\_number\_contig-100\_247.248SRS019601\_contig\_number\_31272SRS019685\_contig\_number\_38427SRS022609\_contig\_number\_45094SRS047014\_contig\_number\_28519SRS054590\_contig\_number\_1752SRS054956\_contig\_number\_14983SRS104400\_contig\_number\_contig-100\_485.341519SRS104636\_contig\_number\_30143SRS1055056\_contig\_number\_contig-100\_226.66826SRS142712\_contig\_number\_3519SRS147139\_contig\_number\_32905SRS147919\_contig\_number\_contig-100\_442.117309SRS893341\_contig\_number\_8875

family
541000
1

1
39492
species

SRS146813\_contig\_number\_11377
